# Development of an electronic interface for transfer of antimicrobial administration data in dairy farms

**DOI:** 10.1371/journal.pone.0278267

**Published:** 2022-12-14

**Authors:** Uwe Seibt, Peter Klötzer, Fanny Rachidi, Uwe Truyen, Stephanie Speck, Alexander Starke, Jil Waade, Walther Honscha

**Affiliations:** 1 Faculty of Veterinary Medicine, Institute of Pharmacology, Pharmacy and Toxicology, Leipzig University, Leipzig, Germany; 2 Faculty of Veterinary Medicine, Clinic for Ruminants and Swine, Leipzig University, Leipzig, Germany; 3 Faculty of Veterinary Medicine, Institute of Animal Hygiene and Veterinary Public Health, Leipzig University, Leipzig, Germany; University of Illinois Urbana-Champaign College of Veterinary Medicine, UNITED STATES

## Abstract

Surveillance of antimicrobial administration in livestock production is an important factor in global policies to reduce spreading of antimicrobial resistance. In recent years, many studies have been carried out concerning the usage of antimicrobials in animal production and in some countries recording of antimicrobial quantities dispensed to famers is mandatory. On cattle farms, antimicrobial treatments are recorded for fattening calves under 8 months of age and for fattening cattle older than 8 months in Germany and treatment frequencies are then calculated. However, with the entry into force of Regulation (EU) 2019/6 on 01/28/2022, antimicrobial monitoring will gradually be extended to all animal species and age groups. Therefore, an effective, fast and accurate transfer of data on the use of antimicrobials, especially in the field of livestock farming, into corresponding databases is required to determine the treatment frequencies for the individual animal species or types of use. For this purpose, an electronic interface was programmed to transfer the data on antimicrobial use in dairy cattle farms from a herd management software program directly into a database. To test the practicability and effectiveness of this interface, 10 dairy cattle farms from Saxony were initially selected. Based on an in-depth analysis of the treatment frequencies of antimicrobial administration of 7 different age groups of animals after a two-year observation period, the functionality of the electronic interface could be established. The greatest potential for reduction of antimicrobials is in newborn calves, as they represent the age group with the highest treatment frequency.

## Introduction

Minimization of antimicrobial administration and a prudent use of these drugs in human and veterinary medicine are necessary to reduce the spreading of antimicrobial resistance (AMR) [1]. Since the last decade a growing global recognition of the importance of AMR and the need for antimicrobial stewardship in human and veterinary medicine became more and more obvious and various national and international bodies developed action plans for AMR reduction [1–6]. According to these plans, countries developed different surveillance systems for monitoring antimicrobial sales and treatment data as reviewed by Sanders et al., 2020 [7]. Outcomes of these studies were published periodically [8–11]. A direct comparison of the antimicrobial use in different countries or of various studies within one country is often hampered by the fact, that the systems differ in many ways, including the kind of data collected, the data source, the type of analysis and the respective output [7]. For harmonization of the studies, the European Medicine Agency (EMA) developed a concept paper on the reporting of antimicrobial sales and usage in animals at the EU level and respective guidelines were published [12, 13]. Most countries implemented antimicrobial monitoring systems on a legal basis. In Germany national sales data of veterinary antimicrobial products were collected and reported to the EUROPEAN Surveillance of Veterinary Antimicrobial Consumption (ESVAC) [11]. In addition, farmers in Germany are required to record treatment data of the food-producing animals. With these data treatment frequencies at farm level are calculated and used as a benchmark. The latest values for treatment frequencies were published in 2022 [14]. A major drawback of this benchmarking system is that antimicrobial treatment records from fattening animals only are collected. For cattle farms, this means that only treatments for fattening calves under 8 months and for fattening cattle older than 8 months are determined. For identification of the reduction potential in administration of antimicrobials on cattle farms an in depth-analysis is mandatory but missing in the literature.

With the entry into force of Regulation (EU) 2019/6 [15] at the beginning of this year, the gradual expansion of the animal species and types of use to be monitored with regard to antimicrobial administration was laid down in law. Therefore, it will be necessary to create a fast and effective procedure to transfer data on antimicrobial treatments, especially from livestock farms, into databases for further evaluation. In Europe, a lack of infrastructure for exchanging this data, as well as different formats and information content of the data, impede the exchange of data between the systems involved [7]. The aim of the study was therefore firstly to develop an electronic interface for the rapid transfer of the relevant data from the herd management program to a database for the calculation of antimicrobial treatment frequencies on dairy farms. In previous studies, animals were often divided only into two age groups (calves, adult animals) [16, 17] or antimicrobial administration was determined at the herd level [18–20]. This allows only a very rough overview of antimicrobial administration in dairy herds. Hence, secondly, for a more detailed analysis of antimicrobial administration, the treatment frequencies in seven different age and use groups in the farms should also be determined in this study.

In the study we successfully developed an electronic interface for transferring antimicrobial administration data in dairy cattle from a herd management program to a database for evaluation of treatment frequencies. In this paper we present the treatment frequencies on a yearly base for the different animal groups, for the different antimicrobial classes, according their applications forms and for the most important indications on dairy farms. The results allow a very detailed overview of the treatment frequencies of seven different age and use groups in dairy herds.

## Material and methods

### Study population

At an information event held as part of a training seminar for dairy farmers in Saxony, the project was presented in detail by the authors, the benefits of the research project were justified and participation by the farms was solicited. The project was to develop a specially programmed interface for the transfer of relevant data from a farm management program to a database for the calculation of antimicrobial treatment frequencies. The functionality of the interface was to be tested in a two-year pilot study. Since the treatment frequencies of all animals of the respective farms were to be determined, the animals were divided into 7 groups according to age and type of use. The animals of each farm were divided into newborn calves (1^st^ to 2^nd^ week), calves (3^rd^ week to 5^th^ month), heifers (6^th^ to 12^th^ month), heifers (13^th^ month to 1^st^ calving), dairy cows, male cattle (6^th^ to 12^th^ month) and male cattle greater than or equal to 13^th^ month.

At the end of the event, ten dairy farms in Saxony in the region around Chemnitz volunteered to participate in the study and confirmed their participation by written consent (respondent-driven sampling). The participating farms mainly kept animals of the German Holstein breed. The dairy farms are approximately representative for Saxony and were randomly selected with the exception that farms use the herd management program “Herde” (Data Service Paretz GmbH, Ketzin, Germany). The herd management program contains detailed information on, among other things, the health, breeding and milk production of each individual animal as well as the entire animal population of the respective farm. Furthermore, the program is multi-user and network-compatible, so that different employees of the farms (herd managers, milkers, veterinarians) can access the data. In addition to manual data entry, a connection to other programs, for example, to control feeding technology, fertility management, various oestrus and activity measurement systems or milking technology providers is also possible.

Descriptive statistics of the main characteristics of the farms and the number of animals per age group are shown in Tables 1 and 2. The data for each individual farm are shown in S1 and S2 Tables.

**Table 1:** Descriptive statistics of the main farm characteristics.

	Minimum	25% Percentile	Median	75% Percentile	Maximum	Mean	Lower 95% CI of mean	Upper 95% CI of mean
**Herd turnover rate (%)**	29	33	34	38	44	35	32	38
**Milk yield (kg/305 days)**	8,294	8,736	9,555	10,517	10,566	9,603	8,939	10,266
**Lifetime production (kg)**	17,995	24,512	28,055	33,827	37,408	28,528	23,885	33,171
**Average tank somatic cell count (x 1,000 cells/ml milk)**	138	177	202	271	325	221	176	267

CI: Lower and upper limit of the 95% confidence interval.

**Table 2:** Descriptive statistics of the average number of animals on the participating farms per age group.

	Minimum	25% Percentile	Median	75% Percentile	Maximum	Mean	Lower 95% CI of mean	Upper 95% CI of mean
**Newborn calves 1st– 2nd week**	12	29.0	35	40.5	86	37.3	27.8	46.8
**Calves 3rd week– 5th month**	22	41.0	162	178.5	458	149.1	86.4	211.7
**Heifers 6th– 12th month**	0	36.0	186	244.5	545	183.1	99.5	266.6
**Heifers 13th month– 1st calving**	39	102.5	340	429.5	893	330.0	200.2	459.8
**Dairy cows**	327	655.5	752	974.5	1,701	837.6	639.3	1,036
**Male cattle 6th– 12th month**	0	0.0	1	21.0	40	10.3	2.5	18.1
**Male cattle ≥ 13th month**	0	0.5	2	16.5	35	8.4	2.3	14.4
**Total**	644	809	1,466	1,844	3,694	1,555.7	1,085	2,027

CI: Lower and upper limit of the 95% confidence interval.

### Data collection

Based on previous studies [21–23] a special online data sheet was developed for collection of the antimicrobial treatment data as part of the web-based VAbData (Veterinary Antibiotics Data) database. This database contains data of all antimicrobial veterinary drugs with a market authorization in Germany. Both, the online data sheet as well as the VAbData database are in German and tailored for Germany only as different drugs are on the market in different countries and there is often a different legal situation. Although the data sheet as well as the database are tailored to German needs, the underlying principle can easily be transferred to other systems used in other countries. The relational database system (DBMS) MySQL was used as the database. The programming of the DBMS and the tools required for administration (backend) and use (frontend) was implemented on the basis of current standards and libraries (including HTML, JavaScript, CSS, PHP). For the direct transfer from the herd management program “Herde”, an interface to Microsoft Excel (Microsoft Corporation, Redmond, USA) was developed for exporting the treatment data. Initially, the programming of an additional module integrated into the herd program for data export was considered but this way proved to be too costly after internal clarifications. The interface uses a VBA macro to access the dBase databases of the herd management program via ODBC functions and transfers the derived data to an Excel spreadsheet. It has been extensively tested as well as selectively adapted and is available online in the OpARA repository. Furthermore, a script was programmed for importing the data from Excel into the VAbData database. Every single antimicrobial administration of an animal, entered by the farmer or his employees, was recorded together with the farm identification number and the animal identification number which allows analysis at both farm and animal level. According to statutory requirements for data protection farmers and location of the farms were anonymized. Extensive functions for data checking and plausibility control have been integrated into this import script.

In principle, the data is checked in a multi-stage process (completeness, correct data type and values, correct drug names, e.g.). Different checks for plausibility followed (diagnosis, disease diagnosis code [24], application form, units e.g.). In the case of questions, farmers were contacted directly and discrepancies clarified, if possible. Individual treatments per animal are considered to belong together and are combined into one record in the database if the animal identification number, antimicrobial drug, disease type, and disease diagnosis code are the same and the time interval between individual drug administrations is less than or equal three days. All different individual administrations with one antimicrobial within the therapy of a disease are therefore combined into one data set.

### Antimicrobial administration data

Antimicrobial administration data were analyzed by drug classes, WHO classification [25], mode of application and diagnosis code [24] for the different age groups. The evaluation of antimicrobial use for the indication diarrhoea in calves includes the diagnoses diarrhoea, calf diarrhoea, enteritis, coli dysentery, coccidiosis and cryptosporidosis. The indication pneumonia in calves includes the diagnoses bronchopneumonia, calf flu and pneumonia. Amounts of active ingredient of the individual antimicrobials were calculated by multiplying the volume or the amounts of tablets administered to the animal by the concentration of the antimicrobial active substance resulting in the total mass of the active ingredient. Drugs whose active ingredients are stated in International Units have been converted to mg/ml or mg/tablets. The conversion factor is usually indicated on the package leaflet of the drug.

Treatment frequencies per year were calculated according the German drug law [26] with minor modifications as shown below.


$$\mathrm{Treatment}\;\mathrm{frequency}\;\mathrm{per}\;\mathrm{year}=\frac{\sum \left(\mathrm{treatment}\;\mathrm{days}\times \mathrm{number}\;\mathrm{of}\;\mathrm{different}\;\mathrm{antimicrobials}\right)}{\sum \mathrm{animal}\;\mathrm{days}\;\mathrm{at}\;\mathrm{risk}/\mathrm{number}\;\mathrm{of}\;\mathrm{days}\;\mathrm{per}\;\mathrm{one}‐\mathrm{year}\;\mathrm{period}}$$

The treatment frequency thus indicates the average number of treatment days per animal in a one-year period. For better comparability, the treatment frequencies of the individual animal groups were normalized to one year. The number of treated animals in the numerator is always 1 per data set. To calculate the average number of animals kept during the one-year period in the denominator, the sum of the animal days at risk per age group was taken from the management software program “Herde”, a program frequently used on dairy farms. No additional treatment days were included for antimicrobials used as one-shot preparations (including long-acting preparations, this refers to drugs that are applied only once but maintain the active level for many days), as the share of these antimicrobials on the farms was low.

### Statistical analysis

Descriptive statistics were performed by using Sigmaplot 14.5 (Systat Software Inc, San Jose, CA, USA), GraphPad 4.0 (GraphPad Software, San Diego, CA, USA) and Excel 2016/2019 (Microsoft Corporation, Redmond, WA, USA). Some of the treatment frequencies are presented as boxplots. The boundary of the box closest to zero indicates the 25^th^ percentile, a line within the box marks the median, and the boundary of the box farthest from zero indicates the 75^th^ percentile. Whiskers (error bars) above and below the box indicate the 90^th^ and 10^th^ percentiles. In addition, the mean is shown as dashed line and outliers as open circles (5^th^ and 95^th^ percentiles).

## Results

Of the total of 10 farmers who had originally declared their written consent to participate in the study, the data from farm 8 (referred to as F8) could not be included in the evaluation because a “0” was entered as the dose in more than 1000 data records. Due to a change in staff, the dose originally used could no longer be determined. In another farm (F10), only the data of the second one-year period could be included in the database. For the remaining farms, the data for each individual treatment of an animal in a herd with antimicrobials could be transferred to the database retrospectively at the start of the study in 2017. One year after the first data collection, the data of the second one-year period (2018–2019) were collected in the same way in order to compensate for annual differences (e.g., epidemic, special weather conditions). In total 131,301 plausible and complete data records of the individual treatments were transferred to the database. 1646 data records (1.24% of all data records) had to be discarded because they were incomplete or could not be reconstructed. Parallel to the transfer of the data sets on antimicrobial use from the project farms, tools were programmed to provide the participating farms with timely online analysis results on farm-specific antimicrobial use per age group (in g per active ingredient, g per antimicrobial class, number of individual treatments per antimicrobial class, average dosages in mg/kg). The output could also be narrowed down in terms of diseases, mode of application and time period. The treatment frequency was calculated according to the formula given in the material and methods section. The treatment frequency thus indicates the average number of treatment days per animal with antimicrobials in the one-year period for the respective age group.

**Fig 1:**
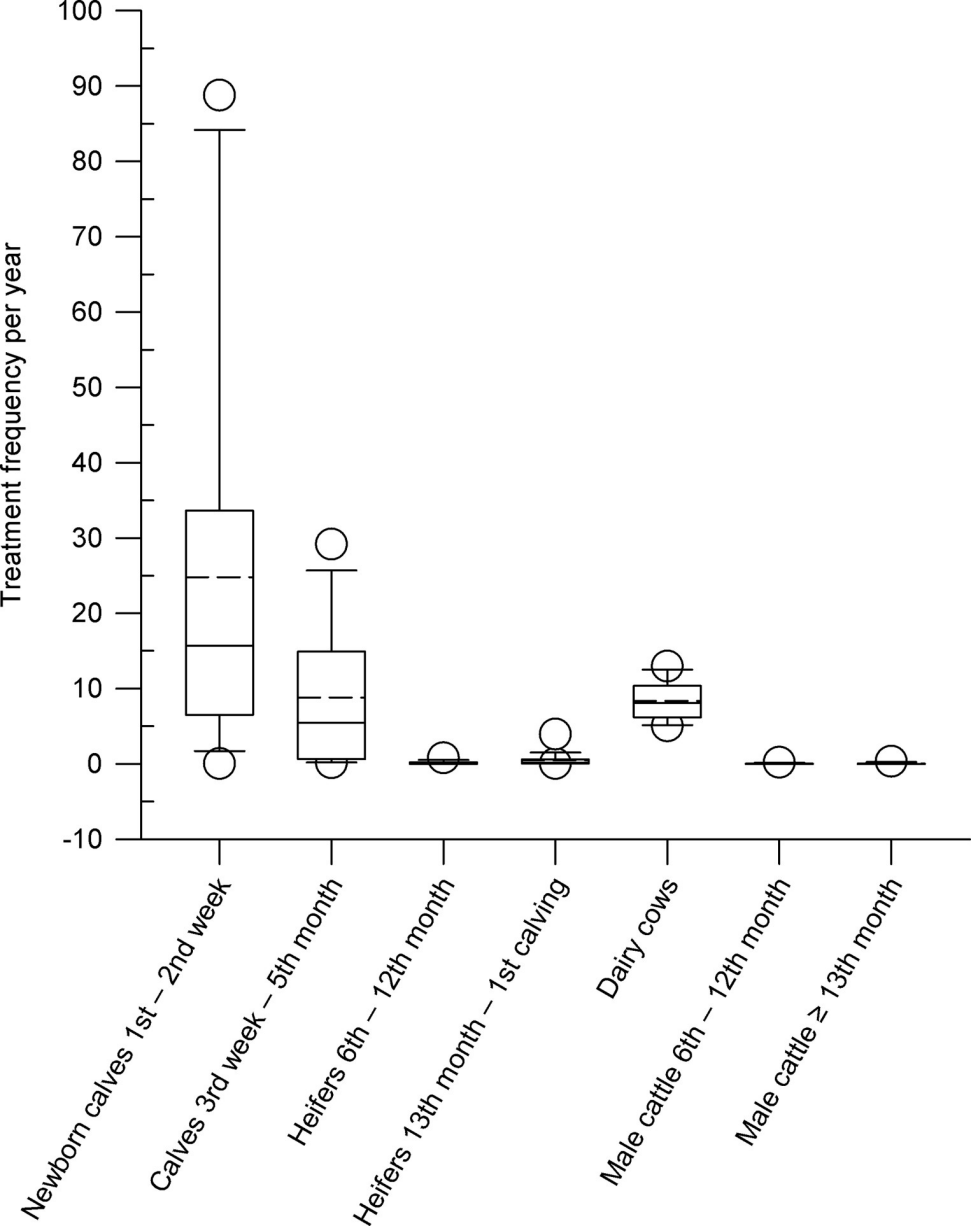
Treatment frequencies of the different age groups per animal and year on nine dairy farms. Box, the range between the 1^st^ (Q1) and 3^rd^ (Q3) quartile; horizontal line, median; dashed line, mean; circles, outlying points (5^th^, 95^th^ percentiles, respectively).

The average treatment frequencies per year in the different age groups of the animals on the farms are shown in Fig 1. Newborn calves show the highest frequency of treatments in the first two weeks of life with a median of 15.65 (mean: 24.82; range 0.03–88.76). This age group of animals had the highest variation of the treatment frequencies. Calves (3^rd^ week to 5^th^ month), on the other hand, are treated much less frequently (median: 5.42; mean: 8.82, range 0.11–29.16). The median frequency of treatments in dairy cows was 8.05 with a mean value of 8.35 (range 4.99–12.93). Treatment frequencies for heifers from 6 to 12 months of age as well as for heifers from 13 months of age to 1^st^ calving were low (median: 0.04, mean: 0.14, range 0–0.79 and median: 0.14, mean: 0.47, range: 0.01–3.91, respectively). Low treatment frequencies were also found for male cattle from 6 to 12 months of age and male cattle from 13 months of age. For male cattle from 6 to 12 months of age, the median was 0 (mean: 0.03, range: 0–0.17). For male cattle 13 months and older, the median frequency of treatments was also 0 (mean: 0.03, range: 0–0.03).

**Fig 2:**
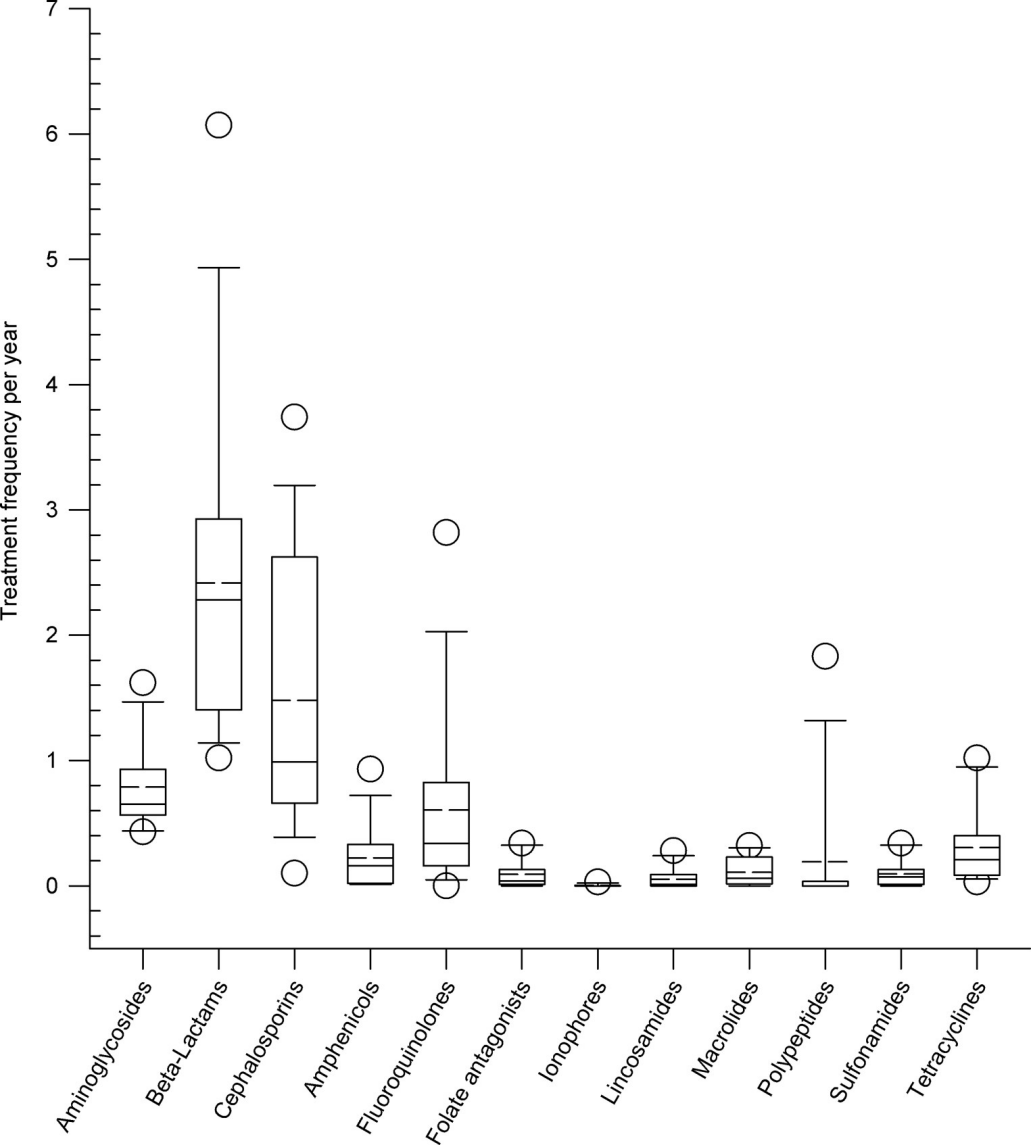
Treatment frequencies of different antimicrobial classes expressed per animal and year on nine dairy farms. Box, the range between the 1^st^ (Q1) and 3^rd^ (Q3) quartile; horizontal line, median; dashed line, mean; circles, outlying points (5^th^, 95^th^ percentiles, respectively).

Beta-lactams (median: 2.28, mean: 2.42, range: 1.02–6.07), cephalosporins (median: 0.99, mean: 1.48, range: 0.10–3.74), aminoglycosides (median: 0.65; mean: 0.79, range: 0.43–1.62) and fluoroquinolones (median: 0.34, mean: 0.61, range: 0–2.82) followed by tetracyclines (median: 0.21, mean: 0.30, range: 0.03–1.02) were the antimicrobial classes with the highest treatment frequencies as shown in Fig 2. Interestingly, pleuromutilins were not used on the farms and folate antagonists, ionophores, lincosamides, polypeptides, and sulfonamides only to a minor extent. A detailed statistical analysis of the administration of different antimicrobial classes per age group is given in S3 Table.

In 2019 the WHO published a classification of antimicrobials accordingly their importance for human medicine [25]. Table 3 shows the antimicrobial classes used on the farms according the WHO classification. Descriptive statistics of the treatment frequencies of the different age groups of the farms enrolled in this study are shown in Table 4. In general, heifers and male cattle were treated only with low frequencies regardless their age. If at all, highly important antimicrobials were used for the treatment of male cattle of both age groups with maximum values of 0.12 (male cattle 6^th^– 12^th^ month) and 0.30 (male cattle older than 13^th^ month). Similar results were received for heifers of both age groups (maximum: 0.68 and 1.70, respectively). In addition to that, heifers of both age groups (heifers 6^th^– 12^th^ month and heifers 13^th^– 1^st^ calving) were treated with high priority critically important antimicrobials (maximum: 0.35 and 2.21, respectively). In descending order, highest priority critically important (median: 3.68, mean: 11.72, range: 0.03–77.64), high priority critically important (median: 3.36, mean: 5.13, range: 0–21.08) and highly important antimicrobials (median: 1.83, mean: 7.77, range: 0–27.99) were used for the therapy of newborn calves (1^st^ and 2^nd^ week). This age group of animals also had the highest variation of the treatment frequencies. Calves from the third week to the fifth month, on the other hand, were mainly treated with highly important antimicrobials (median 3.00, mean: 3.70, range: 0.03–10.29) followed by highest priority critically and high priority critically important antimicrobials. Dairy cows were mainly treated with highly important antimicrobials (median: 3.26, mean: 3.87, range: 2.28–6.24), high priority critically important antimicrobials (median: 1.89, mean: 2.27, range: 0.90–5.07) and highest priority critically important antimicrobials (median: 1.69, mean: 2.20, range: 0.28–5.71).

**Table 3:** Antimicrobial classes used according the WHO classification.

	Antimicrobial classes used on nine dairy farms
**Highest Priority Critically Important Antimicrobials**	Cephalosporins (3rd, 4th and 5th generation), Fluoroquinolones, Macrolides, Polypeptides
**High Priority Critically Important Antimicrobials**	Aminoglycosides (without Spectinomycin), Penicillins (Aminopenicillins)
**Highly Important Antimicrobials**	Cephalosporins (1st and 2nd generation), Amphenicols, Folate antagonists, Lincosamides, Penicillins (anti-staphylococcal, narrow spectrum), Sulfonamides, Tetracyclines
**Important Antimicrobials**	Aminocyclitols (Spectinomycin)

**Table 4:** Descriptive statistics of the treatment frequencies per year of the different age groups according the WHO classification.

		Minimum	25% Percentile	Median	75% Percentile	Maximum	Mean	Lower 95% CI of mean	Upper 95% CI of mean
**Highest Priority Critically Important Antimicrobials**									
	Newborn calves 1st– 2nd week	0.03	0.52	3.68	9.49	77.64	11.72	0.27	23.17
	Calves 3rd week– 5th month	0.03	0.25	1.31	7.36	14.24	3.81	0.96	6.66
	Heifers 6th– 12th month	0	0	0	0.04	0.09	0.02	0.00	0.03
	Heifers 13th month– 1st calving	0	0	0.02	0.05	0.07	0.03	0.01	0.04
	Dairy cows	0.28	0.94	1.69	2.91	5.71	2.20	1.35	3.04
	Male cattle 6th– 12th month	0	0	0	0	0.05	0.01	0.00	0.01
	Male cattle ≥ 13th month	0	0	0	0	0.06	0.00	0.00	0.01
**High Priority Critically Important Antimicrobials**									
	Newborn calves 1st– 2nd week	0	0.09	3.36	10.05	21.08	5.13	1.92	8.34
	Calves 3rd week– 5th month	0	0.04	0.77	2.30	6.17	1.28	0.41	2.14
	Heifers 6th– 12th month	0	0	0	0.02	0.35	0.04	-0.01	0.09
	Heifers 13th month– 1st calving	0	0	0.01	0.08	2.21	0.17	-0.10	0.44
	Dairy cows	0.90	1.55	1.89	2.94	5.07	2.27	1.70	2.84
	Male cattle 6th– 12th month	0	0	0	0	0.08	0.01	0.00	0.02
	Male cattle ≥ 13th month	0	0	0	0	0	0	0	0
**Highly Important Antimicrobials**									
	Newborn calves 1st– 2nd week	0	0.57	1.83	17.93	27.99	7.77	2.55	12.98
	Calves 3rd week– 5th month	0.03	0.44	3.00	6.75	10.29	3.70	1.88	5.52
	Heifers 6th– 12th month	0	0	0.02	0.05	0.68	0.09	-0.01	0.18
	Heifers 13th month– 1st calving	0.01	0.03	0.06	0.49	1.70	0.27	0.05	0.50
	Dairy cows	2.28	2.91	3.26	4.84	6.24	3.87	3.23	4.51
	Male cattle 6th– 12th month	0	0	0	0	0.12	0.01	-0.01	0.03
	Male cattle ≥ 13th month	0	0	0	0	0.30	0.03	-0.01	0.08
**Important Antimicrobials**									
	Newborn calves 1st– 2nd week	0	0	0	0	2.47	0.19	-0.13	0.51
	Calves 3rd week– 5th month	0	0	0	0	0.28	0.03	-0.01	0.07
	Heifers 6th– 12th month	0	0	0	0	0.01	0.00	0.00	0.00
	Heifers 13th month– 1st calving	0	0	0	0	0	0	0	0
	Dairy cows	0	0	0	0	0	0	0	0
	Male cattle 6th– 12th month	0	0	0	0	0	0	0	0
	Male cattle ≥ 13th month	0	0	0	0	0	0	0	0

0 means that no treatments took place, while 0.00 means that the number of treatments was so low that the value was rounded to 0.00. CI: Lower and upper limit of the 95% confidence interval.

**Fig 3:**
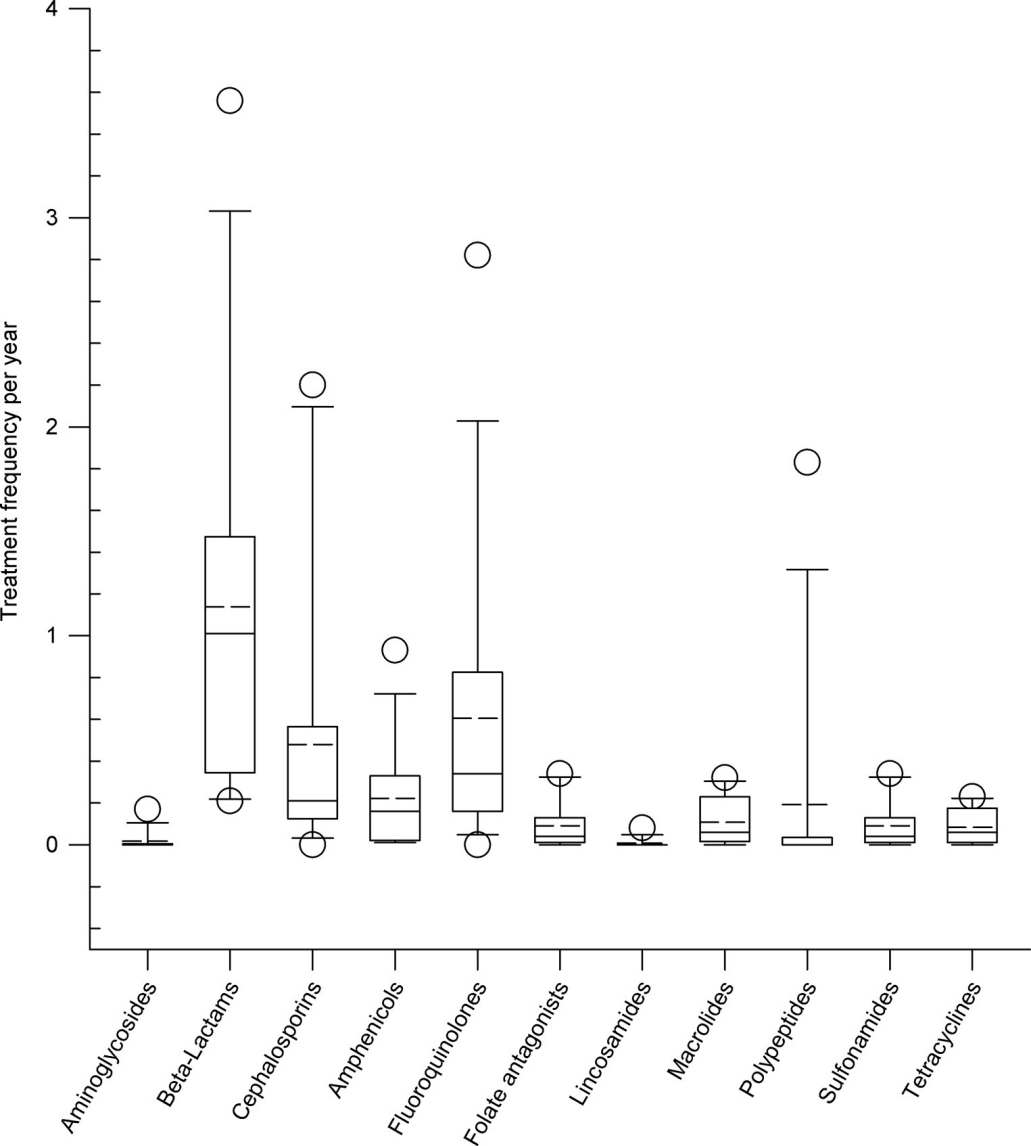
Administration of different antimicrobial classes after parenteral application expressed as treatment frequencies per animal and year on nine dairy farms. Box, the range between the 1^st^ (Q1) and 3^rd^ (Q3) quartile; horizontal line, median; dashed line, mean; circles, outlying points (5^th^, 95^th^ percentiles, respectively).

Beta-lactams, fluoroquinolones and cephalosporins were most commonly used for parenteral therapy of cattle on farms, while aminoglycosides, lincosamides and polypeptides were very rarely used (Fig 3).

As expected, antimicrobials were rarely administered orally on the farms. Only active substances from the groups of aminoglycosides, beta-lactams and ionophores were used here. Therefore, the median was zero or around zero.

**Fig 4:**
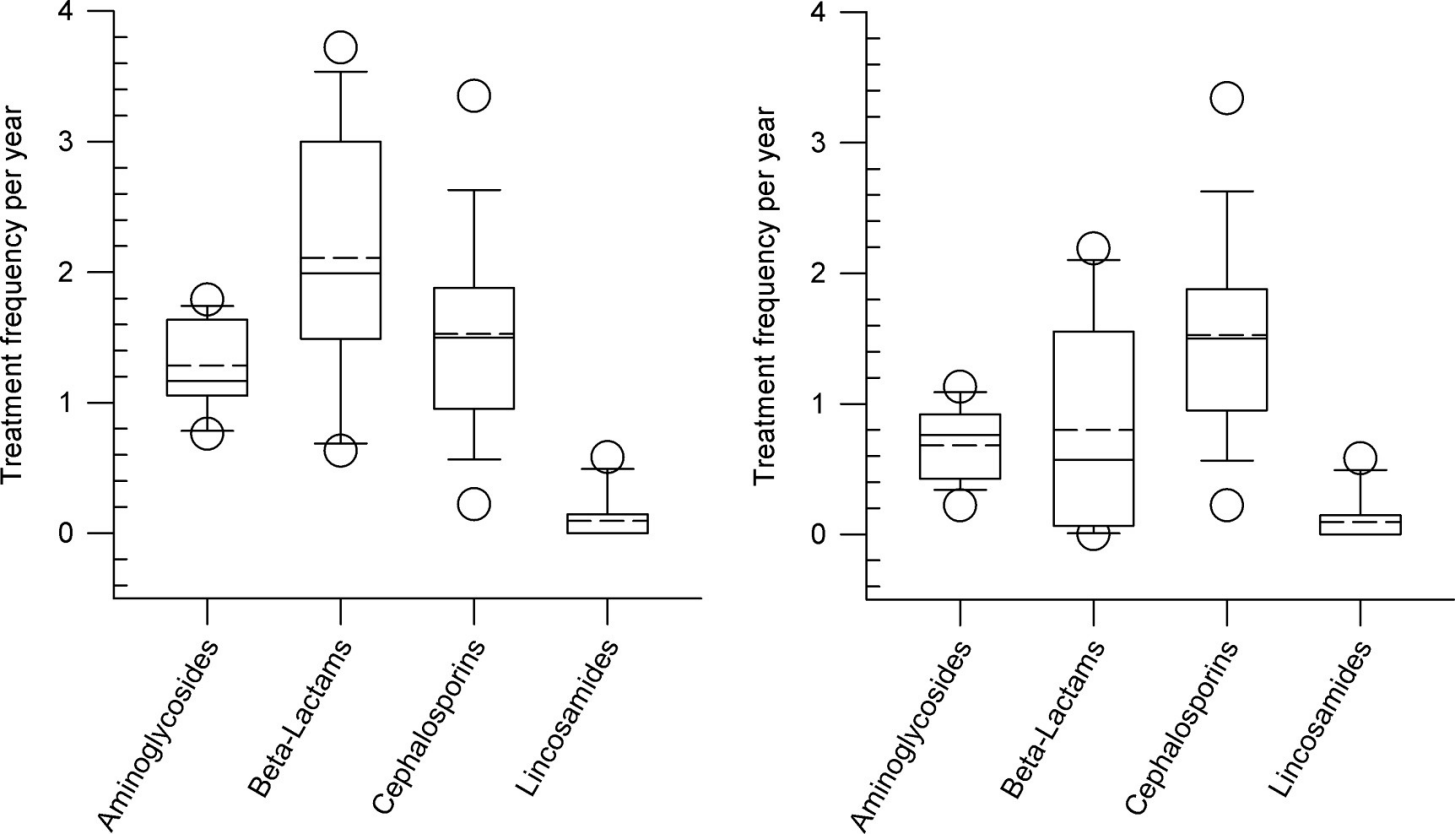
Administration of different antimicrobial classes after intramammary application expressed as treatment frequencies per animal and year on nine dairy farms. A: Intramammary application including dry-cow therapy (DCT), B: Intramammary application without DCT. Box, the range between the 1^st^ (Q1) and 3^rd^ (Q3) quartile; horizontal line, median; dashed line, mean; circles, outlying points (5^th^, 95^th^ percentiles, respectively).

Annual treatment frequencies of the different classes of antimicrobials for intramammary use with and without dry-cow therapy (DCT) are shown in Fig 4A and 4B. Beta-lactames, cephalosporins, aminoglycosides and lincosamides were used for intramammary application including DCT (Fig 4A). With the exception of lincosamides, the other groups of active substances mentioned above have also been used in rare cases (treatment frequencies between 0.01 and 0.02) in the therapy of heifers (13^th^ month– 1^st^ calving). For intramammary therapy without DCT, cephalosporins, aminoglycosides, beta-lactams and lincosamides were used in descending order (Fig 4B).

For intrauterine therapy of dairy cows tetracyclines, beta-lactams and cephalosporins were used in descending order (Table 5). Heifers (13^th^ month to 1^st^ calving) were also treated with tetracyclines (median: 0, mean: 0.00, range: 0–0.01) and beta-lactams (median: 0, mean: 0.00, range: 0–0.05).

**Table 5:** Descriptive statistics of the antimicrobial classes used for intrauterine application of dairy cows.

	Minimum	25% Percentile	Median	75% Percentile	Maximum	Mean	Lower 95% CI of mean	Upper 95% CI of mean
**Tetracyclines**	0	0.02	0.06	0.44	0.96	0.24	0.08	0.40
**Beta-lactams**	0	0	0.04	0.16	0.52	0.09	0.02	0.16
**Cephalosporins**	0	0	0.01	0.03	0.07	0.02	0.00	0.03

0 means that no treatments took place, while 0.00 means that the number of treatments was so low that the value was rounded to 0.00. CI: Lower and upper limit of the 95% confidence interval.

Finally, the treatment frequencies of important diseases in dairy farms were determined. The focus was on, pneumonia, enteritis and udder diseases.

Table 6 shows that newborn calves in particular were treated with antimicrobials against pneumonia in the first two weeks (median: 4.24, mean: 5.47, range: 0–18.08), followed by calves from the third week of life up to the fifth month (median: 3.91, mean: 5.36, range: 0.10–20.18). In the latter age group, however, treatment of enteritis plays a much smaller role than in newborn calves (median: 0.23, mean: 0.76, range: 0–5.76 versus 3.46, 5.02, 0–14.88, respectively). Udder diseases requiring treatment naturally occur most frequently in dairy cows, but heifers are also treated with antimicrobials to a small extent.

**Table 6:** Descriptive statistics of the treatment frequencies per year of the different age groups for different indications.

		Minimum	25% Percentile	Median	75% Percentile	Maximum	Mean	Lower 95% CI of mean	Upper 95% CI of mean
**Pneumonia**									
	Newborn calves 1st– 2nd week	0	1.58	4.24	8.60	18.08	5.47	2.94	8.01
	Calves 3rd week– 5th month	0.10	0.46	3.91	8.20	20.18	5.36	2.39	8.33
	Heifers 6th– 12th month	0	0	0	0.02	0.15	0.02	0.00	0.04
	Heifers 13th month– 1st calving	0	0	0	0.01	0.08	0.01	0.00	0.02
	Dairy cows	0	0	0.02	0.23	0.85	0.15	0.02	0.29
	Male cattle 6th– 12th month	0	0	0	0	0.08	0.01	0.00	0.02
	Male cattle ≥ 13th month	0	0	0	0	0.15	0.01	-0.01	0.03
**Enteritis**									
	Newborn calves 1st– 2nd week	0	0.46	3.46	10.30	14.88	5.02	2.29	7.75
	Calves 3rd week– 5th month	0	0.07	0.23	0.62	5.76	0.76	0.01	1.52
	Heifers 6th– 12th month	0	0	0	0	0.01	0.00	0.00	0.00
	Heifers 13th month– 1st calving	0	0	0	0	0.02	0.00	0.00	0.00
	Dairy cows	0	0	0.01	0.03	0.08	0.02	0.00	0.03
	Male cattle 6th– 12th month	0	0	0	0	0	0	0	0
	Male cattle ≥ 13th month	0	0	0	0	0	0	0	0
**Udder diseases including IDCT** ^**1**^									
	Newborn calves 1st– 2nd week	0	0	0	0	0	0	0	0
	Calves 3rd week– 5th month	0	0	0	0	0	0	0	0
	Heifers 6th– 12th month	0	0	0	0	0	0	0	0
	Heifers 13th month– 1st calving	0	0	0	0.02	0.03	0.01	0.00	0.01
	Dairy cows	3.40	4.40	5.42	8.56	9.06	6.16	5.12	7.21
	Male cattle 6th– 12th month	0	0	0	0	0	0	0	0
	Male cattle ≥ 13th month	0	0	0	0	0	0	0	0
**Udder diseases without IDCT** ^**1**^									
	Newborn calves 1st– 2nd week	0	0	0	0	0	0	0	0
	Calves 3rd week– 5th month	0	0	0	0	0	0	0	0
	Heifers 6th– 12th month	0	0	0	0	0	0	0	0
	Heifers 13th month– 1st calving	0	0	0	0.02	0.03	0.01	0.00	0.01
	Dairy cows	1.18	3.00	3.53	6.28	7.11	4.24	3.31	5.18
	Male cattle 6th– 12th month	0	0	0	0	0	0	0	0
	Male cattle ≥ 13th month	0	0	0	0	0	0	0	0

0 means that no treatments took place, while 0.00 means that the number of treatments was so low that the value was rounded to 0.00. ^1^IDCT: Intramammary dry-cow therapy. CI: Lower and upper limit of the 95% confidence interval.

## Discussion

Responsible use of antimicrobials in both human and veterinary medicine is essential to reduce the further spread of antimicrobial resistance. For this reason, various international institutions (WHO, EFSA, EMA) have pointed out the importance of this global problem and developed various action plans and strategies for the prudent use of antimicrobials. A number of nations have implemented different monitoring and surveillance programs, the results of which are published regularly. In addition to the collection of sales data of antimicrobials to veterinarians in Germany [10, 11], the quantification of antimicrobial administration in veterinary medicine is particularly crucial in order to measure effects of minimisation strategies on farms and to enable benchmarking [7, 27]. Currently, however, in accordance with the German drug law, antimicrobial monitoring is only mandatory in Germany for fattening animals (cattle, pigs, chickens, turkeys). Antimicrobial use in other food-producing animals such as dairy cattle, calves, and male cattle not intended for fattening, and heifers is not recorded. In January 2022 Regulation (EU) 2019/6 [15] came into force, which gradually requires antimicrobial use to be recorded not only for fattening animals, but for all food-producing animals.

The aim of the study was therefore firstly to develop an electronic interface between the herd management program and a database for the evaluation of antimicrobial usage and to optimize the interface within the project in order to simplify data transfer and to avoid manual transfer of data. Since the functionality of the interface was to be tested first and as further modifications might be required, only a smaller number of farms were initially selected for the study. Secondly, antimicrobial administration in dairy herds of all animals kept on the farms, i.e. also animals not intended for fattening, were to be recorded. For this in-depth analysis, the animals were divided into 7 different age groups and type of use, since an effective reduction of antimicrobial administration is only possible with detailed knowledge of the individual age groups.

It turned out that the use of an electronic interface has great advantages over other methods of data entry [18]. Transmission errors in the manual entry of application and delivery documents are practically eliminated, provided that correct documentation is done in the herd management program. Furthermore, this method is very fast and the individual treatments of each animal in the respective herd are recorded. This is, for example, not possible with the so-called garbage can method [18, 19, 28] and the use of application and delivery receipts as a data basis, is very time-consuming [21, 22]. Other authors used questionnaires sent to livestock keepers as a data base to determine antimicrobial administration [20, 29–31] and likewise sales data [32] served as a basis. The use of electronic interfaces to government databases on antimicrobial administration [17, 33] or to practice software [34, 35] occurs rather less frequently. However, as the number of different software programs for veterinary practices is much larger, an interface to a herd management program was developed that can be used almost universally. Furthermore, a herd management program offers a much higher density of information, as additional data on each animal is collected (milk yield, etc.), allowing for a very detailed analysis of the farm.

In a study from the Netherlands, data on antimicrobial use and dispensing were transferred from the practice management system to a central database [34]. However, the current weight of treated animals was not recorded and the authors used standardized animal weights for the different types of use to calculate the treatment frequencies. This poses the risk in the case of fast-growing young animals such as newborn calves that the treatment frequencies calculated in this way may be inaccurate, as pointed out by Bokma et al, 2019 [36]. In this paper [36], treatment data from the veterinarian’s practice software were transferred to an Excel spreadsheet and treatment frequencies were calculated using either standard weights or current weights of treated animals determined from standard growth curves. The frequencies calculated in this way differed by a factor of 2.5.

To circumvent this problem, the exact age of the treated animals was recorded in our study when the data were transferred using the electronic interface, thus assigning the animals to the different age groups. Since the age groups were closely staggered, a relatively accurate indication of the average weight of the treated animals was possible.

As reviewed by Sanders et al., 2020 [7], the currently existing monitoring systems were described and it was shown that it has unfortunately not yet been possible to harmonize the evaluation of antimicrobial administration and thus arrive at comparable key indicators. For example, there is a multitude of different formulas for the calculation of treatment frequencies in which frequently estimated variables for the weight of the animals at the time of treatment or standardized doses (e.g. defined daily dose animal, DDDA) are used to calculate the treatment days from the administration quantity of the respective antimicrobial. Since the treatment frequencies calculated in this way are based on these variables, the values are subject to a certain degree of inaccuracy. This is particularly noticeable in young animals that gain a lot of body weight in a short time. To circumvent these problems, the number of treatment days and the number of active substances used per treatment were set in relation to the number of days the animals were kept in the respective age group in order to calculate the treatment frequency per year. Such count-based indicators were used in this or similar ways in Austria, Canada, Switzerland, Germany, Finland, France, Sweden and Great Britain and served for benchmarking [7]. However, these calculations often differ in the denominator used in the equation. For example, some of the indicators were calculated on total animal capacity [16] or per production cycle [7], making a direct comparison of treatment frequencies difficult.

Ten dairy farms in Saxony were enrolled in the study via convenience sampling. A total of 131,301 data records were transferred from the herd management program of the participating farms via interface to the database and evaluated in the years 2017 to 2019. The treatment frequencies per year of the farms for the individual age classes of the animals are shown in Fig 1. While heifers and male cattle show only very low treatment frequencies regardless of the age class (median between 0.04 and 0.14 for heifers and 0.0 for male cattle), the highest treatment frequencies were determined in descending order for newborn calves, lactating cows and calves from the third week of life to the 5^th^ month. The greatest variability among farms was found for newborn calves (0.03 and 88.76 as min and max values, respectively). For calves from the 3^rd^ week of life up to the 5^th^ month, a certain variability in treatment frequencies of the farms was observed in comparison to all other age groups. At the same time, it becomes clear that the greatest reduction potential with regard to antimicrobial use lies with newborn calves. Measures to reduce antimicrobial administration should therefore focus on this age group. Since in the majority of other studies antimicrobial administration in calves was considered over a longer period of time [17, 21, 22, 30, 34], this resulted in significantly lower indicators for treatment frequency. Antimicrobial usage data (AMU) for veal calves were published by Bokma et al, 2019 and 2020 [36, 37]. The mean AMU values were 32.3 ± 11.04 and 30.1 ± 10.4 defined daily doses for animals per year, respectively.

The most frequently used classes of active ingredients on the farms were, in descending order, beta-lactames, cephalosporins, aminoglycosides, fluroquinolones and tetracyclines (Fig 2). While Lardé et al, 2021 [27] and Saini et al, 2012 [18] came to different conclusions, Stevens et al, 2018 [19] found a broadly similar distribution of antimicrobial classes on dairy farms. Active ingredients from the antimicrobial classes cephalosporins, penicillins, tetracyclines, and trimethoprim/sulfonamides, on the other hand, predominated in Saini et al, 2012 [18], while Lardé et al, 2021 [27] described ionophores, penicillins, aminocoumarins, and aminoglycosides as the most frequently used groups of antimicrobial agents. These differences in the use of antimicrobial classes in different countries can be explained in part by differences in licensure requirements. For example, from the group of ionophores, only Monensin A has a market authorization in Germany.

In Table 3 it can be seen that newborn calves have the highest treatment frequencies of all age groups for highest priority critically important antimicrobials (cephalosporins 3^th^ to 5^th^ generation, macrolides and quinolones) and high priority antimicrobials (aminoglycosides and aminopenicillins). This makes even more obvious how important it is to record antimicrobial administration in this age group in particular. Therefore, especially in this age group, increased efforts should be made to reduce the administration of antimicrobials through improved husbandry and hygiene conditions as well as intensive care of the animals. Highly important antimicrobials like 1^st^ and 2^nd^ generation cephalosporins and narrow spectrum penicillins, on the other hand, are used primarily in dairy cows and calves (3^rd^ week– 5^th^ month). Antimicrobials from the group of important antimicrobials are rarely used. The frequent use of cephalosporins (3^rd^ and 4^th^ generation) in dairy farms was also described by Saini et al. [18] in Canada and also in Belgium, according to Stevens et al [19, 28], a very high proportion of dairy cattle are treated with 4^th^ generation cephalosporins.

Respiratory tract diseases (pneumonia) and enteritis are the most common diseases in young calves [20, 30, 31, 38]. As can be seen from Table 6, pneumonia requiring treatment occurred in both newborn calves and calves up to 5 months of age. Digestive tract diseases (enteritides) were treated with antimicrobials mainly in newborn calves and less in older calves. In agreement with Bos et al. [34], the age group of newborn calves showed particularly high treatment frequencies. However, this age group included animals up to 98 days of age [34].

Fig 1 shows that next to newborn calves (median 15.65) dairy cows (8.05) have the second highest frequency of treatments. The majority of antimicrobial use in dairy cows is for the treatment of udder disease. The median treatment frequency in dairy cows with udder disease including intramammary dry cow therapy (IDCT) is 5.42, while the median for udder disease without IDCT is 3.53. In summary, it can be stated that in dairy farms antimicrobials are mainly applied parenterally and intramammary, while per os administration or intrauterine application are only rarely performed. These results are in agreement with data from Kuipers et al. [39]. In this study from the Netherlands, 68% of the total animal defined daily dosage was used to treat udder disease on dairy farms. For intramammary therapy, mainly agents from the groups of beta-lactams, cephalosporins and aminoglycosides were used. According to Cuong et al. [40] penicillin, ceftiofur, ampicillin and sulfonamides are mainly used in cattle farms. However, in the farms studied here, only a very low treatment frequency for sulfonamides was found.

## Conclusion

Herd management programs are often used in dairy farms. Since this software stores not only all information on the individual animals in the herd, such as age and type of use, but also all antimicrobial treatments, they are an ideal source for determining the treatment frequencies of antimicrobial use.

Therefore, an electronic interface between a herd management program and a special database for the collection and analysis of antimicrobial administration data was developed. The functionality and practicability of the interface were tested successfully on initially 10 Saxon dairy farms over a period of two years. The quality and correctness of data entry as well as the success in the development of the electronic interface and the import script is shown by the very low percentage of data sets that could not be used for evaluation. This concerned only 1.24% of all data records. The animals of the individual farms were stratified into a total of 7 groups according to age and type of use. In previous studies, antimicrobial use in calves was often observed over a longer period of time and comparatively moderate treatment frequencies were determined. However, in this study, the highest treatment frequencies were determined for the first time in newborn calves up to 14 days of age. Calves from 3 weeks to 5 months of age, on the other hand, already showed significantly lower treatment frequencies. These data indicate that newborn calves should be given special consideration in minimization concepts for the use of antimicrobials in dairy farms, since this age group offers the highest potential for savings.

## Supporting information

S1 Table Farm characteristics of each farm. (DOCX)

S2 Table Number of animals in the different age and use groups per farm in the first and second observation period. (DOCX)

S3 Table Descriptive statistics of the treatment frequencies of different antimicrobial classes per age and use group. (DOCX)
