# Supplementary material for: Development of an electronic interface for transfer of antimicrobial administration data in dairy farms
Source: PLoS One. 2022 Dec 14;17(12):e0278267. doi: 10.1371/journal.pone.0278267 (PMC9749987; doi:10.1371/journal.pone.0278267)
Supplement: S1 Table — (DOCX) [file pone.0278267.s001.docx]

| Farm | F1 | F2 | F3 | F4 | F5 | F6 | F7 | F9 | F10 |
| --- | --- | --- | --- | --- | --- | --- | --- | --- | --- |
| Herd turnover rate (%) | 33 | 37 | 38 | 34 | 44 | 34 | 33 | 29 | 32 |
| Milk yield (kg/305 days) | 10,531 | 10,566 | 9,535 | 8,692 | 8,780 | 8,294 | 10,503 | 9,969 | 9,555 |
| Lifetime production (kg) | 37,408 | 36,031 | 28,055 | 25,535 | 17,995 | 23,488 | 27,431 | 29,183 | 31,623 |
| Average tank somatic cell count (x 1,000 cells/ml milk) | 202 | 255 | 197 | 180 | 234 | 287 | 138 | 174 | 325 |

**Table S1: Farm characteristics of each farm**

The data from farm 8 (F8) could not be included in the evaluation.
