# Supplementary material for: Development of an electronic interface for transfer of antimicrobial administration data in dairy farms
Source: PLoS One. 2022 Dec 14;17(12):e0278267. doi: 10.1371/journal.pone.0278267 (PMC9749987; doi:10.1371/journal.pone.0278267)
Supplement: S2 Table — (DOCX) [file pone.0278267.s002.docx]

**Table S2:** **Number of animals in the different age and use groups per farm in the first and second observation period.**

| Farm | F1.1 | F1.2 | F2.1 | F2.2 | F3.1 | F3.2 | F4.1 | F4.2 | F5.1 | F5.2 | F6.1 | F6.2 | F7.1 | F7.2 | F9.1 | F9.2 | F10.2 |
| --- | --- | --- | --- | --- | --- | --- | --- | --- | --- | --- | --- | --- | --- | --- | --- | --- | --- |
| Newborn calves 1st – 2nd week | 40 | 39 | 29 | 29 | 34 | 35 | 31 | 32 | 39 | 38 | 14 | 12 | 73 | 86 | 41 | 44 | 18 |
| Calves 3rd week – 5th month | 161 | 178 | 26 | 27 | 22 | 23 | 162 | 173 | 180 | 179 | 67 | 55 | 390 | 458 | 163 | 168 | 102 |
| Heifers 6th – 12th month | 194 | 236 | 0 | 0 | 0 | 0 | 165 | 186 | 264 | 252 | 80 | 72 | 520 | 545 | 223 | 237 | 138 |
| Heifers 13th month – 1st calving | 443 | 456 | 55 | 61 | 49 | 39 | 340 | 293 | 416 | 387 | 172 | 144 | 893 | 856 | 387 | 383 | 236 |
| Dairy cows | 921 | 940 | 662 | 649 | 741 | 752 | 710 | 695 | 834 | 821 | 350 | 327 | 1,698 | 1,701 | 1,031 | 1,009 | 399 |
| Male cattle 6th – 12th month | 1 | 1 | 0 | 0 | 0 | 0 | 24 | 40 | 0 | 1 | 18 | 15 | 36 | 38 | 0 | 0 | 1 |
| Male cattle ≥ 13th month | 2 | 2 | 0 | 0 | 0 | 0 | 34 | 35 | 2 | 2 | 19 | 19 | 14 | 10 | 1 | 1 | 1 |
| Total | 1,762 | 1,852 | 772 | 766 | 846 | 849 | 1,466 | 1,454 | 1,735 | 1,680 | 720 | 644 | 3,624 | 3,694 | 1,846 | 1,842 | 895 |

F1.1: Farm 1, one-year period 1; F1.2: Farm 1, one-year period 2; etc. The data from farm 8 (F8) could not be included in the evaluation. From farm 10 (F10), only the data of the second one-year period could be included.
