# Supplementary material for: Development of an electronic interface for transfer of antimicrobial administration data in dairy farms
Source: PLoS One. 2022 Dec 14;17(12):e0278267. doi: 10.1371/journal.pone.0278267 (PMC9749987; doi:10.1371/journal.pone.0278267)
Supplement: S3 Table — (DOCX) [file pone.0278267.s003.docx]

**Table S3. Descriptive statistics of the treatment frequencies of different antimicrobial classes per age and use group.**

|  |  | Minimum | 25% Percentile | Median | 75% Percentile | Maximum | Mean | Std. Deviation | Std. Error | Lower 95% CI of mean | Upper 95% CI of mean |
| --- | --- | --- | --- | --- | --- | --- | --- | --- | --- | --- | --- |
| Aminoglycosides | Newborn calves 1st – 2nd week | 0 | 0 | 0 | 0.05 | 5.50 | 0.59 | 1.52 | 0.37 | -0.19 | 1.37 |
|  | Calves 3rd week – 5th month | 0 | 0 | 0 | 0.27 | 1.10 | 0.15 | 0.30 | 0.07 | 0.00 | 0.30 |
|  | Heifers 6th – 12th month | 0 | 0 | 0 | 0 | 0.01 | 0.00 | 0.00 | 0.00 | 0.00 | 0.00 |
|  | Heifers 13th month – 1st calving | 0 | 0 | 0 | 0.01 | 0.01 | 0.00 | 0.00 | 0.00 | 0.00 | 0.00 |
|  | Dairy cows | 0.80 | 1.06 | 1.17 | 1.64 | 1.79 | 1.29 | 0.33 | 0.08 | 1.12 | 1.46 |
|  | Male cattle 6th – 12th month | 0 | 0 | 0 | 0 | 0 | 0 | 0 | 0 | 0 | 0 |
|  | Male cattle ≥ 13th month | 0 | 0 | 0 | 0 | 0 | 0 | 0 | 0 | 0 | 0 |
|  | Total | 0.43 | 0.57 | 0.65 | 0.93 | 1.62 | 0.79 | 0.33 | 0.08 | 0.62 | 0.96 |
|  |  |  |  |  |  |  |  |  |  |  |  |
| Beta-Lactams | Newborn calves 1st – 2nd week | 0 | 1.31 | 4.41 | 14.33 | 24.14 | 8.25 | 7.91 | 1.92 | 4.19 | 12.32 |
|  | Calves 3rd week – 5th month | 0 | 0.10 | 1.52 | 3.13 | 5.73 | 1.91 | 2.04 | 0.50 | 0.86 | 2.96 |
|  | Heifers 6th – 12th month | 0 | 0 | 0 | 0.04 | 0.36 | 0.05 | 0.10 | 0.02 | 0.00 | 0.09 |
|  | Heifers 13th month – 1st calving | 0 | 0.01 | 0.05 | 0.38 | 3.60 | 0.38 | 0.87 | 0.21 | -0.07 | 0.82 |
|  | Dairy cows | 1.77 | 2.33 | 2.77 | 4.95 | 5.94 | 3.45 | 1.43 | 0.35 | 2.72 | 4.19 |
|  | Male cattle 6th – 12th month | 0 | 0 | 0 | 0 | 0.08 | 0.01 | 0.02 | 0.00 | 0.00 | 0.02 |
|  | Male cattle ≥ 13th month | 0 | 0 | 0 | 0 | 0 | 0 | 0 | 0 | 0 | 0 |
|  | Total | 1.02 | 1.41 | 2.28 | 2.93 | 6.07 | 2.42 | 1.34 | 0.33 | 1.73 | 3.11 |
|  |  |  |  |  |  |  |  |  |  |  |  |
| Cephalosporins | Newborn calves 1st – 2nd week | 0 | 0 | 0 | 0.55 | 4.04 | 0.52 | 1.08 | 0.26 | -0.04 | 1.08 |
|  | Calves 3rd week – 5th month | 0 | 0 | 0 | 0.15 | 4.33 | 0.70 | 1.53 | 0.37 | -0.09 | 1.49 |
|  | Heifers 6th – 12th month | 0 | 0 | 0 | 0 | 0.03 | 0.00 | 0.01 | 0.00 | 0.00 | 0.01 |
|  | Heifers 13th month – 1st calving | 0 | 0 | 0.01 | 0.04 | 0.06 | 0.02 | 0.02 | 0.00 | 0.01 | 0.03 |
|  | Dairy cows | 0.22 | 1.33 | 1.72 | 2.89 | 5.56 | 2.33 | 1.49 | 0.36 | 1.57 | 3.10 |
|  | Male cattle 6th – 12th month | 0 | 0 | 0 | 0 | 0 | 0 | 0 | 0 | 0 | 0 |
|  | Male cattle ≥ 13th month | 0 | 0 | 0 | 0 | 0.06 | 0.00 | 0.01 | 0.00 | 0.00 | 0.01 |
|  | Total | 0.10 | 0.66 | 0.99 | 2.63 | 3.74 | 1.48 | 1.11 | 0.27 | 0.91 | 2.05 |
|  |  |  |  |  |  |  |  |  |  |  |  |
| Amphenicols | Newborn calves 1st – 2nd week | 0 | 0.26 | 1.18 | 1.74 | 6.83 | 1.57 | 1.83 | 0.44 | 0.63 | 2.51 |
|  | Calves 3rd week – 5th month | 0.03 | 0.27 | 1.45 | 4.24 | 6.56 | 2.23 | 2.25 | 0.55 | 1.07 | 3.39 |
|  | Heifers 6th – 12th month | 0 | 0 | 0 | 0.01 | 0.05 | 0.01 | 0.01 | 0.00 | 0.00 | 0.01 |
|  | Heifers 13th month – 1st calving | 0 | 0 | 0 | 0 | 0.00 | 0 | 0 | 0 | 0 | 0 |
|  | Dairy cows | 0 | 0 | 0 | 0 | 0 | 0 | 0 | 0 | 0 | 0 |
|  | Male cattle 6th – 12th month | 0 | 0 | 0 | 0 | 0.04 | 0.00 | 0.01 | 0.00 | 0.00 | 0.01 |
|  | Male cattle ≥ 13th month | 0 | 0 | 0 | 0 | 0 | 0 | 0 | 0 | 0 | 0 |
|  | Total | 0.01 | 0.02 | 0.16 | 0.33 | 0.93 | 0.22 | 0.25 | 0.06 | 0.09 | 0.35 |
|  |  |  |  |  |  |  |  |  |  |  |  |
| Fluoroquinolones | Newborn calves 1st – 2nd week | 0 | 0.02 | 0.47 | 7.95 | 46.31 | 6.54 | 11.66 | 2.83 | 0.54 | 12.53 |
|  | Calves 3rd week – 5th month | 0 | 0.07 | 0.23 | 3.40 | 8.15 | 1.83 | 2.95 | 0.72 | 0.31 | 3.35 |
|  | Heifers 6th – 12th month | 0 | 0 | 0 | 0.01 | 0.02 | 0.00 | 0.01 | 0.00 | 0.00 | 0.01 |
|  | Heifers 13th month – 1st calving | 0 | 0 | 0 | 0 | 0.01 | 0.00 | 0.00 | 0.00 | 0.00 | 0.00 |
|  | Dairy cows | 0.01 | 0.28 | 0.39 | 0.70 | 1.65 | 0.49 | 0.41 | 0.10 | 0.28 | 0.70 |
|  | Male cattle 6th – 12th month | 0 | 0 | 0 | 0 | 0 | 0 | 0 | 0 | 0 | 0 |
|  | Male cattle ≥ 13th month | 0 | 0 | 0 | 0 | 0 | 0 | 0 | 0 | 0 | 0 |
|  | Total | 0.00 | 0.16 | 0.34 | 0.83 | 2.82 | 0.61 | 0.74 | 0.18 | 0.23 | 0.98 |
|  |  |  |  |  |  |  |  |  |  |  |  |
| Folate antagonists | Newborn calves 1st – 2nd week | 0 | 0 | 0 | 0.52 | 9.82 | 1.17 | 2.96 | 0.72 | -0.35 | 2.69 |
|  | Calves 3rd week – 5th month | 0 | 0 | 0.01 | 0.15 | 1.45 | 0.19 | 0.41 | 0.10 | -0.02 | 0.40 |
|  | Heifers 6th – 12th month | 0 | 0 | 0 | 0.01 | 0.05 | 0.00 | 0.01 | 0.00 | 0.00 | 0.01 |
|  | Heifers 13th month – 1st calving | 0 | 0 | 0 | 0.01 | 0.02 | 0.00 | 0.01 | 0.00 | 0.00 | 0.01 |
|  | Dairy cows | 0 | 0.02 | 0.08 | 0.15 | 0.29 | 0.09 | 0.08 | 0.02 | 0.05 | 0.13 |
|  | Male cattle 6th – 12th month | 0 | 0 | 0 | 0 | 0 | 0 | 0 | 0 | 0 | 0 |
|  | Male cattle ≥ 13th month | 0 | 0 | 0 | 0 | 0 | 0 | 0 | 0 | 0 | 0 |
|  | Total | 0 | 0.01 | 0.04 | 0.13 | 0.34 | 0.09 | 0.11 | 0.03 | 0.03 | 0.15 |
|  |  |  |  |  |  |  |  |  |  |  |  |
| Lincosamides | Newborn calves 1st – 2nd week | 0 | 0 | 0 | 0 | 2.47 | 0.19 | 0.62 | 0.15 | -0.13 | 0.51 |
|  | Calves 3rd week – 5th month | 0 | 0 | 0 | 0 | 0.28 | 0.03 | 0.08 | 0.02 | -0.01 | 0.07 |
|  | Heifers 6th – 12th month | 0 | 0 | 0 | 0 | 0.01 | 0.00 | 0.00 | 0.00 | 0.00 | 0.00 |
|  | Heifers 13th month – 1st calving | 0 | 0 | 0 | 0 | 0 | 0 | 0 | 0 | 0 | 0 |
|  | Dairy cows | 0 | 0 | 0 | 0.15 | 0.58 | 0.09 | 0.18 | 0.04 | 0.00 | 0.19 |
|  | Male cattle 6th – 12th month | 0 | 0 | 0 | 0 | 0 | 0 | 0 | 0 | 0 | 0 |
|  | Male cattle ≥ 13th month | 0 | 0 | 0 | 0 | 0 | 0 | 0 | 0 | 0 | 0 |
|  | Total | 0 | 0 | 0.01 | 0.09 | 0.28 | 0.05 | 0.09 | 0.02 | 0.01 | 0.10 |
|  |  |  |  |  |  |  |  |  |  |  |  |
| Macrolides | Newborn calves 1st – 2nd week | 0 | 0.02 | 0.07 | 0.32 | 4.38 | 0.45 | 1.06 | 0.26 | -0.10 | 0.99 |
|  | Calves 3rd week – 5th month | 0 | 0.12 | 0.49 | 0.89 | 1.57 | 0.56 | 0.49 | 0.12 | 0.31 | 0.82 |
|  | Heifers 6th – 12th month | 0 | 0 | 0 | 0.01 | 0.08 | 0.01 | 0.02 | 0.01 | 0.00 | 0.02 |
|  | Heifers 13th month – 1st calving | 0 | 0 | 0 | 0 | 0.05 | 0.01 | 0.02 | 0.00 | 0.00 | 0.01 |
|  | Dairy cows | 0 | 0 | 0 | 0.12 | 0.47 | 0.09 | 0.17 | 0.04 | 0.00 | 0.18 |
|  | Male cattle 6th – 12th month | 0 | 0 | 0 | 0 | 0.05 | 0.01 | 0.02 | 0.00 | 0.00 | 0.01 |
|  | Male cattle ≥ 13th month | 0 | 0 | 0 | 0 | 0 | 0 | 0 | 0 | 0 | 0 |
|  | Total | 0 | 0.02 | 0.06 | 0.23 | 0.32 | 0.11 | 0.12 | 0.03 | 0.05 | 0.17 |
|  |  |  |  |  |  |  |  |  |  |  |  |
| Polypeptides | Newborn calves 1st – 2nd week | 0 | 0 | 0 | 0.43 | 41.75 | 4.22 | 11.69 | 2.84 | -1.79 | 10.23 |
|  | Calves 3rd week – 5th month | 0 | 0 | 0 | 0.63 | 4.23 | 0.72 | 1.45 | 0.35 | -0.03 | 1.46 |
|  | Heifers 6th – 12th month | 0 | 0 | 0 | 0 | 0 | 0 | 0 | 0 | 0 | 0 |
|  | Heifers 13th month – 1st calving | 0 | 0 | 0 | 0 | 0 | 0 | 0 | 0 | 0 | 0 |
|  | Dairy cows | 0 | 0 | 0 | 0 | 0 | 0 | 0 | 0 | 0 | 0 |
|  | Male cattle 6th – 12th month | 0 | 0 | 0 | 0 | 0 | 0 | 0 | 0 | 0 | 0 |
|  | Male cattle ≥ 13th month | 0 | 0 | 0 | 0 | 0 | 0 | 0 | 0 | 0 | 0 |
|  | Total | 0 | 0 | 0 | 0.04 | 1.83 | 0.19 | 0.51 | 0.12 | -0.07 | 0.45 |
|  |  |  |  |  |  |  |  |  |  |  |  |
| Sulfonamides | Newborn calves 1st – 2nd week | 0 | 0 | 0 | 0.52 | 9.82 | 1.17 | 2.96 | 0.72 | -0.35 | 2.69 |
|  | Calves 3rd week – 5th month | 0 | 0 | 0.01 | 0.15 | 1.45 | 0.19 | 0.41 | 0.10 | -0.02 | 0.40 |
|  | Heifers 6th – 12th month | 0 | 0 | 0 | 0.01 | 0.05 | 0.00 | 0.01 | 0.00 | 0.00 | 0.01 |
|  | Heifers 13th month – 1st calving | 0 | 0 | 0 | 0.01 | 0.02 | 0.00 | 0.01 | 0.00 | 0.00 | 0.01 |
|  | Dairy cows | 0 | 0.02 | 0.11 | 0.16 | 0.29 | 0.10 | 0.08 | 0.02 | 0.05 | 0.14 |
|  | Male cattle 6th – 12th month | 0 | 0 | 0 | 0 | 0 | 0 | 0 | 0 | 0 | 0 |
|  | Male cattle ≥ 13th month | 0 | 0 | 0 | 0 | 0 | 0 | 0 | 0 | 0 | 0 |
|  | Total | 0 | 0.01 | 0.07 | 0.13 | 0.34 | 0.10 | 0.11 | 0.03 | 0.04 | 0.15 |
|  |  |  |  |  |  |  |  |  |  |  |  |
| Tetracyclines | Newborn calves 1st – 2nd week | 0 | 0 | 0 | 0.07 | 1.63 | 0.16 | 0.41 | 0.10 | -0.06 | 0.37 |
|  | Calves 3rd week – 5th month | 0 | 0 | 0.06 | 0.53 | 1.56 | 0.31 | 0.45 | 0.11 | 0.07 | 0.54 |
|  | Heifers 6th – 12th month | 0 | 0 | 0 | 0.02 | 0.59 | 0.06 | 0.17 | 0.04 | -0.02 | 0.15 |
|  | Heifers 13th month – 1st calving | 0 | 0 | 0.03 | 0.08 | 0.31 | 0.06 | 0.08 | 0.02 | 0.01 | 0.10 |
|  | Dairy cows | 0.04 | 0.12 | 0.40 | 0.55 | 1.03 | 0.40 | 0.30 | 0.07 | 0.24 | 0.55 |
|  | Male cattle 6th – 12th month | 0 | 0 | 0 | 0 | 0.12 | 0.01 | 0.03 | 0.01 | -0.01 | 0.03 |
|  | Male cattle ≥ 13th month | 0 | 0 | 0 | 0 | 0.30 | 0.03 | 0.09 | 0.02 | -0.01 | 0.08 |
|  | Total | 0.03 | 0.09 | 0.21 | 0.40 | 1.02 | 0.30 | 0.29 | 0.07 | 0.16 | 0.45 |

0 means that no treatments took place, while 0.00 means that the number of treatments was so low that the value was rounded to 0.00.
